# In Vivo Effects of A Pro-PO System Inhibitor on the Phagocytosis of *Xenorhabdus Nematophila* in *Galleria Mellonella* Larvae

**DOI:** 10.3390/insects10090263

**Published:** 2019-08-22

**Authors:** Andrea De Lerma Barbaro, Marzia B. Gariboldi, Maristella Mastore, Maurizio F. Brivio, Stefano Giovannardi

**Affiliations:** 1 Laboratory of Comparative Physiopathology, Department of Biotechnology and Life Sciences, University of Insubria, 21052 Busto Arsizio (Varese), Italy; 2 Laboratory of Anticancer Pharmacology, Department of Biotechnology and Life Sciences, University of Insubria, 21052 Busto Arsizio (Varese), Italy; 3 Laboratory of Comparative Immunology and Parasitology, Department of Theoretical and Applied Sciences, University of Insubria, 21100 Varese, Italy

**Keywords:** immunomodulatory drugs, in vitro vs in vivo, opsonization, phagocytosis escape, proPO system, *Xenorhabdus nematophila*

## Abstract

*Xenorhabdus nematophila* is a Gram-negative bacterium symbiont of the entomopathogen nematode *Steinernema carpocapsae* whose immunosuppressive properties over host’s immune response have been thoroughly investigated. In particular, live *X. nematophila* actively impairs phagocytosis in host’s hemocytes through the secretion of inhibitors of eicosanoids synthesis. In this article we have investigated the cell surface structural features of *X. nematophila* responsible for the elusion from phagocytosis. To this end we have studied the uptake of heat-killed (hk), fluorescein isothiocyanate (FITC)-labeled *X. nematophila* by phagocytes from both a host insect and a mammalian species. In vitro dead *X. nematophila* passively resists engulfment by insect hemocytes without impairing the phagocytosis machinery whereas, unexpectedly, in vivo a significant phagocytosis of dead *X. nematophila* was observed. *X. nematophila* in vivo phagocytosis was increased by the co-injection of the specific inhibitor of pro-phenoloxidase (PO) system phenylthiourea (PTU), even if these effects were not observed in in vitro tests. Furthermore, biochemical modifications of *X. nematophila* cell wall implement in vivo phagocytosis, suggesting that this bacterium avoid phagocytosis because the ligand of phagocytic receptors is somehow buried or disguised in the cell wall. Finally, dead *X. nematophila* escapes engulfment even by human phagocytes suggesting that *X. nematophila* could be a useful model to investigate escape from phagocytosis by mammalian macrophages.

## 1. Introduction

*Insect immunity, an outline.* Insect innate immune responses may be broadly categorized into humoral and cellular effector mechanisms [1,2]. Humoral immunity involves synthesis of various antibacterial proteins, enzymes such as lysozyme and activation of the pro-phenoloxidase (pro-PO) system. Cellular immunity involves direct contact between circulating hemocytes and the invaders, examples of cellular immunity are phagocytosis and nodulation. Phagocytosis, the internalization and killing of microbes, is the basic cellular defense mechanism against bacteria and fungi [3,4]. Most metazoans feature dedicated, “professional” phagocytes; granular cells and plasmatocytes are the lepidopteran phagocytes [2,4,5]. Nodulation develops as the result of the micro aggregation of hemocytes leading to the entrapment of microbes [6]; nodulation is of utmost relevance in counteracting the spreading of sepsis in the case of massive infection or active bacterial proliferation inside the insect body. Melanization, at the crossroad of humoral and cellular defenses, is a key step in cellular immune responses being involved in both phagocytosis and nodulation [7]. Phenoloxidase (PO) is predominantly synthesized in hemocytes as a zymogen called pro-PO and released into hemolymph by cell rupture, in lepidopterans, proPO is expressed in oenocytoids [2,7]. The released pro-PO is just an inactive zymogen and requires the proteolytic cleavage by pro-PO activating proteases. The activated PO catalyzes a complex cascade of reactions leading to melanin deposition around the invading parasites. Of note, reactive oxygen species (ROS), by products of the melanization cascade, play an important part in sterilization of microorganisms [8]. In addition to melanization, eicosanoids play a pivotal role practically in all the cellular defense functions of insects, including phagocytosis, and nodulation [9,10].

*Phagocytosis, an outline.* Due to the subject of this article, it is worthwhile to introduce some basic knowledge on cell biology of phagocytosis [3]. The first step in phagocytosis is the detection of the particle. Microbial pathogens are recognized directly by receptors that bind pathogen-associated molecular patterns (PAMPs) (phagocytosis opsonin-independent) or indirectly by receptors that bind opsonins (phagocytosis opsonin-dependent). The dedicated cell surface phagocytic receptors that directly bind PAMPs are a subset of pattern recognition receptors (PRRs) and are well characterized in mammals. Among the most studied opsonic phagocytic receptors in mammals are the complement receptors (CRs), interacting with the activated complement fragment C3b. It is worth stressing that in mammals the engulfment of target microorganisms is greatly enhanced after opsonization. Several phagocytic receptors involved in opsonin-independent phagocytosis have been characterized in insects including the model organism *Drosophila melanogaster* [11] and several lepidoptera species [4]. Opsonin-dependent phagocytosis has been demonstrated and studied in molecular details in mosquitos [11] and investigated even in the lepidoptera, *Galleria mellonella*. In the latter species apolipophorin III [12] and GmCP8 [13] act as opsonins enhancing in vitro phagocytic activity of haemocytes. Nevertheless, the overall evidence of opsonin-dependent phagocytosis in insect is lower if compared to opsonin-dependent, for example complement-driven, phagocytosis in mammals.

*Pathogen’s escape from phagocytosis.* Successful pathogenic bacteria and fungi in mammals have evolved multiple strategies to subvert the phagocytosis process [14,15]. Regarding the resistance to the first step of phagocytosis, namely attachment to and internalization in phagocytes, the bacterial surface plays often a key role; pathogenic bacteria and fungi display on cell surface polysaccharide capsules but even specific molecular moieties that obstacle both opsonin-dependent and opsonin independent ingestions. Furthermore, some pathogens may secrete substances that undermine phagocytosis; these molecules act as toxins that disrupt the signaling of phagocytosis or exert a broad cytotoxic effect on hemocytes. Finally, in insects less knowledge about escape from phagocytosis in comparison to mammals is currently available [4,11].

*Xenorhabdus entomopathogenic bacteria, an outline.* In the last three decades, a great deal of research has been done to elucidate the tripartite interaction between entomopathogenic bacteria of the genus *Xenorhabdus*, their nematode vectors of the genus *Steinernema*, and the insect larvae target of infection [16]. In brief, *Xenorhabdus/Steinernema* life cycle can be summarized into three phases: (a) infection of the insect host, (b) bacterial and nematode reproduction and symbiotic re-association and finally (c) transmission to new host. In the hemolymph *Xenorhabdus* bacteria counteracts the host immune system inducing a fatal septicemia along with toxemia to kill the target insect; accordingly, degraded larvae tissues provide a nutrient source for *Xenorhabdus* and its nematode vector. Among *Xenorhabdus* species, *X. nematophila* is the best understood regarding molecular mechanisms of symbiosis and inhibition of host immune functions. *X. nematophila* commonly infects Lepidoptera, many of which are significant agricultural pests. When experimentally injected into larvae, *X. nematophila* is highly virulent, also in the absence of its nematode vector. These bacteria also suppress insect immune responses acting on many aspects of host’s physiology [16,17]; actually, a prominent target is eicosanoid biosynthesis. This effect is due to the active secretion by live bacteria of trans-4-phenyl-3-buten-2-one (BZA) that potently and selectively inhibits the insect phospholipase A2 (PLA2) [9,18]. In connection with the pivotal role of eicosanoids in insect cellular immunity, live but not heat-killed (hk) *X. nematophila* impair phagocytosis and nodulation [19]. *X. nematophila* produces an array of cytolysins/hemolysins and toxins, some of which have been shown to induce necrosis or apoptosis when added to cultures of insect immune cells [20,21,22]; moreover, the bacteria can counteract, at the transcriptional level, the inducible expression of cationic antimicrobial peptides (AMPs), as shown for cecropin [23,24]. Finally, even if not addressed in this issue, it is worth mentioning the direct immunomodulatory and inhibitory effects of the *Steinernema* nematode parasites on their insect hosts [25,26].

*Rationale of the study and experimental design.* As explained above, in most cases the suppression of host immunity by *X. nematophila* is the outcome of the active growth of alive, toxins secreting bacteria inside the host’s body. Indeed, this is the case in *Spodoptera exigua*; in this insect, alive *X. nematophila* impairs phagocytosis in the host’s hemocytes through the secretion of inhibitors of PLA2, a key enzyme required for the synthesis of eicosanoids [27]. In mammal models some pathogenic fungi or bacteria are endowed with an avoidance ability from uptake by professional phagocytes due to cell surface structural intrinsic features, an escape strategy therefore expected in dead bacterial particles as well [14,15]. In this report we have investigated the cell surface features of *X. nematophila* responsible for the elusion from phagocytosis. To this end we have studied the uptake of heat-killed, fluorescein isothiocyanate (FITC)-labeled *X. nematophila* by phagocytes in caterpillars of the natural host *G. mellonella*. In addition, we have studied the encounter between bacteria and phagocytes under different host’ physiological conditions. Firstly, comparing the outcome on phagocytosis of in vitro and in vivo challenge with *X. nematophila* and finally, verifying the uptake of dead *X. nematophila* by a human macrophage cell line, derived from an organism that is in evolutionary terms very distant from insects.

## 2. Material and Methods

### 2.1. Insect Host and Bacterial Strains

As insect host, we used larvae of the wax moth *G. mellonella* reared on a sterile food mixture; only healthy late stage caterpillars (VI instar), 20–25 mm long, 4–5 mm wide and 180–250 mg in weight [28,29], were selected for the experiments. To obtain the symbiont bacterium *X. nematophila,* larvae of *G. mellonella* were infected with infective juvenile (IJ) *Steinernema carpocapsae* (Capsanem^®^ from Koppert Biological Systems, Berkel en Rodenrijs, The Netherlands), and 24 h post infection, dead larvae were surface sterilized in 70% ethanol and air dried. Then, larvae were punctured with needles and drops of hemolymph were streaked with inoculation loops onto nutrient agar plates containing NBTA (40 g/L nutrient tryptone soy agar, 25 mg/L bromothymol blue powder and 40 mg/L 2,3,5 triphenyl tetrazolium chloride) (Sigma-Aldrich, Milan, Italy). Plates were incubated at 28 °C in the dark for 24 h, and then a single colony of bacteria was selected and streaked onto a new plate of NBTA. Sub culturing was continued until colonies of uniform size and morphology, displaying a swarming motility on 0.8 % agar were obtained [30,31]. The 16S rRNA genotyping of our bacterial isolate was carried out by BMR Genomics Padova, Italy; for the experiments *X. nematophila* AN6/1 was used. The virulence of the isolated *X. nematophila* strain was evaluated by infection of *G. mellonella* larvae. The bacterial concentration was estimated by spectrophotometric reading absorbance (λ = 600 nm) and the injection of 10^2^ CFU killed 100% larvae within 33 h (Figure 2C). *E. coli* strain C1α (kindly provided by Dr. Viviana Orlandi, Università dell’Insubria, Varese, Italy) was cultured overnight in lysogeny broth (LB) at 37 °C and the bacterial concentration was estimated by spectrophotometric reading absorbance (λ = 600 nm). Both *X. nematophila* and *E. coli* were killed by incubating phosphate-buffered saline (PBS) suspended bacteria at 68 °C for 10 minutes. Bacterial stocks were stored at −80 °C in LB broth supplemented with 20% (v/v) glycerol.

### 2.2. Bleeding of Insects, Hemocyte Cultures and Separation of Cell-Free Plasma

Before bleeding or injection, larvae were anesthetized by chilling them on ice for 5 min and surface sterilized. Whole hemolymph was collected by cutting the second proleg with sterile micro scissors and drawing the hemolymph into a pre chilled Eppendorf tube containing an approximately equal volume of Mead anticoagulant buffer (98 mM NaOH, 145 mM NaCl, 17 mM ethylenediaminetetraacetic acid (EDTA) and 41 mM citric acid, pH 4.5) (Sigma-Aldrich, Milan, Italy). After cell counting in a Neubauer chamber (in some experiments the viability of hemocytes was assessed by exclusion of trypan blue dye (Merck, Milan, Italy) under a microscope. Sub confluent monolayers were prepared by plating 2 × 10^5^ cells in 24-well polystyrene culture plates (Corning, Amsterdam, The Netherlands) filled with 500 μL phosphate buffered saline PBS (137 mM NaCl, 2.68 mM KCl, 8.10 mM Na_2_HPO_4_, 1.47 mM KH_2_PO_4_, pH 7.4), 2 × 10^5^ hemocytes were contained in 10–20 μL /150–250 μL hemolymph plus Mead buffer. The hemocytes were allowed to adhere and spread for 30 min at 25 °C, then the wells were washed 3 times with PBS to remove non-adherent hemocytes and debris. At last the wells were refilled with 500 μL of lepidoptera medium, Grace’s insect medium (Sigma-Aldrich, Milan, Italy) supplemented with 10% mammalian fetal calf serum (FCS) and 1% antibiotic antimycotic solution (10,000 units penicillin, 10 mg streptomycin and 25 μg amphotericin B per mL) (Sigma-Aldrich, Milan, Italy) for in vitro phagocytosis assay (see below) or in 500 μL of PBS for observation after in vivo phagocytosis assay (see below). Cell free plasma was obtained from whole hemolymph pooled from five caterpillars by several centrifugations performed at 200 g for 10 min at 4 °C in a swing out rotor. To inhibit the activation of the pro-PO system, phenylthiourea (PTU) (Alfa Aesar, Kandel, Germany), solubilized in 1:1 ethanol/PBS, stock solution 50 mM, was added to hemolymph at the final concentration of 1.5 mM. The cleared hemolymph was used immediately for the bacterial opsonization experiments (see below).

### 2.3. Fluorescence Labelling and Cell Surface Biochemical Modifications of Bacteria 

For phagocytosis assays fluorescent-labeled bacteria were used. Cell surface FITC-labeled bacteria were prepared according to [32] by incubating for 1 hour at room temperature in the dark 3 × 10^8^ heat-killed (hk) bacterial cells/mL in 0.1 mg/mL fluorescein isothiocyanate (FITC) (Sigma-Aldrich, Milan, Italy) solubilized in 0.1 M Na_2_CO_3_/NaHCO_3_ carbonate buffer pH 9.0. The stock solution of 2 mg/mL FITC was prepared in dimethyl sulfoxide DMSO (Sigma-Aldrich, Milan, Italy). FITC conjugated bacteria were rinsed three times with PBS to remove unbound FITC, finally resuspended in PBS at the concentration of 10^6^ bacterial cells/μL and stored in aliquots at −20 °C. In some experiments the cell surface of fluorescence-labeled bacteria was modified, before being used in the phagocytosis test, by treatment with proteinase K (Sigma-Aldrich, Milan, Italy) or with urea (Sigma-Aldrich, Milan, Italy). Fluorescent-labeled, heat-killed bacteria 10^6^ cells/μL were pelleted at 800 g, resuspended in an equal volume of a buffer containing 50 mM Tris pH 7.5, 5 mM CaCl_2_ and proteinase K was added at a final concentration of 0.2 or 1.0 μg/mL. After 10 minutes at 23 °C bacteria were pelleted and resuspended in PBS at 10^6^ cells/μL for use in phagocytosis assays. Alternatively, fluorescence-labeled, heat killed bacteria 10^6^ cells/μL were pelleted at 800 g, resuspended in an equal volume of urea 2, 4 or 6M and incubated for 10 minutes at 22 °C. For use in phagocytosis assays bacteria were washed out and resuspended in PBS at 10^6^ cells/μL.

These biochemical modifications of bacterial cell surface produce an experimental bias, the decreased intensity of the fluorescence signal after treatment of FITC-labeled bacteria. In order to address this point, we carried out by FP 750 spectrofluorometer (Jasco, Easton, MD, USA) a quantitative evaluation of detached fluorescence analyzing the supernatants of labeled bacteria after treatments with proteinase K or urea and centrifugation. The measurements indicated that a significant amount of FITC was recovered in supernatant of FITC-labeled bacteria: (a) the fluorescence signal in the supernatant increased with the dose treatment (b) the fluorescence signals were higher in samples after proteinase K digestion in comparison to urea treatment (c) as a whole the fluorescence signals were similar in supernatant from *E. coli*-FITC and *X. nematophila*-FITC (Figure S1). Nevertheless, even after biochemical modification the fluorescence signal remained bright enough for an informative assessment of phagocytosis as shown in the result section. De-frozen bacterial stock were never re-frozen as freezing cycles can compromise the bacterial integrity and introduce a bias in the experimental results.

### 2.4. Phagocytosis Assays

*In vitro phagocytosis assays:* FITC-labeled bacteria were added, at 5 × 10^6^ cells/well, to freshly separated hemocytes cultured in lepidoptera medium at sub confluence, 2 × 10^5^ cells in 24 multi-well plates. This is a calculated bacterium/hemocytes ratio of 25:1. After 2.5 h at 25 °C phagocytosis was evaluated by fluorescence microscopy or, after hemocytes resuspension by treatment with trypsin/EDTA, by flow cytometry. Unless otherwise stated, each experimental point was analyzed independently on hemocytes extracted from five larvae. In some phagocytosis tests of FITC-labeled *E. coli*, different amounts of heat killed or alive *X. nematophila* bacteria, as indicated in results, were added 3 hours before the challenge with fluorescent bacteria. In some in vitro phagocytosis experiments, the test was carried out in the presence of PTU at concentrations ranging from 10 to 90 μM. In some in vitro experiments, FITC-labeled *X. nematophila* was pelleted and resuspended in cell cleared hemolymph for 30 minutes at 22 °C, then hemocytes were challenged with the opsonized FITC-labeled bacteria. 

*In vivo phagocytosis assays:* 10 μL of 1 x 10^6^ cells/μL FITC-labeled bacteria resuspended in PBS were injected in caterpillars using a Hamilton gas tight syringe (Hamilton, Reno, NE, USA) with a 0.30 mm in diameter needle. After 1.5 h at 22 °C, hemocytes were extracted from larvae and plated at sub confluence in 24 multi wells in PBS, then the cells were allowed to adhere for 30 minutes at 25 °C. Phagocytosis was evaluated by fluorescence microscopy or, after detachment by treatment with trypsin/EDTA, by flow cytometry. Unless otherwise stated, each experimental point was replicated independently in five larvae. In in vivo double injection experiments, different amounts of heat killed or alive *X. nematophila* bacteria, as indicated in results, were injected in caterpillars 3 hours before the challenge with FITC-labeled *E. coli*. Otherwise, different amounts of heat-killed *E. coli*, as indicated in results, were injected in caterpillars 24 hours before the challenge with FITC-labeled *X. nematophila*. This procedure, defined as “priming”, was carried out to boost the host’s immune response [33,34]. Finally, in in vivo co-injection phagocytosis experiments, FITC-labeled *X. nematophila* (10^7^ cells in 10 μL) was injected in PBS containing immunomodulatory drugs, 5 mM PTU or 5 μg/μL N-acetyl-cysteine (NAC) (Sigma-Aldrich, Milan, Italy). 

### 2.5. Evaluation of Phagocytosis by Fluorescence Microscopy and by Flow Cytometry

**Figure 1:**
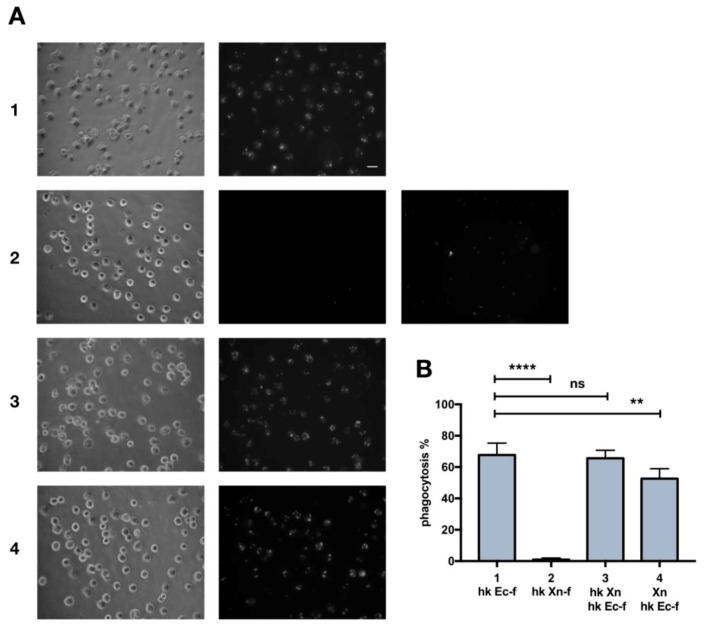
Heat killed X. nematophila in vitro avoids but does not inhibit phagocytosis in insect hemocytes. (**A**) bright field (left panels) and fluorescence after trypan blue addition (middle panels) images, each series is from the same field of hemocytes cell cultures. 1, FITC-labeled, heat killed E. coli (hk Ec-f), fluorescent bacteria added to wells 2.5 h before the observation. 2, FITC-labeled, heat killed X. nematophila (hk Xn-f), fluorescent bacteria added to wells 2.5 h before the observation (right panel is before trypan blue addition). 3 and 4, hemocytes cultures are pre-treated for 3h with 5x106/well not labeled, heat killed (hk Xn) and 5x106/well alive (Xn) X. nematophila respectively; then FITC-labeled E. coli (hk Ec-f) is added and 2.5 h later images were acquired. 1–4, FITC-labeled bacteria added at a calculated bacteria/hemocyte ratio of 25:1. Calibration bar 20 μm. (**B**) cell count statistics acquired from images as depicted in A. Each experimental point was replicated in five larvae, it is shown the statistical analysis from three independent trials (n=15). Percentage of phagocytosis is calculated as ratio between fluorescent cells that display a clear pattern of phagocytosis after trypan blue quenching and total cells in the images, multiplied by 100. Statistical analysis is executed on angular transformed data (see material and methods) two-way ANOVA and Tukey HSD test is applied between pairs of data as indicated by horizontal bars (**** *p* < 0.0001; ** *p*< 0.01).

To discriminate a mere surface adhesion from a real phagocytosis, we used the trypan blue quenching method [35,36]. Trypan blue quenches only the fluorescence associated to bacteria non-phagocytized. Cells adherent on plastic or in suspension were extensively washed in PBS to remove trace of proteins in solution that could reduce the trypan blue quenching effect. Trypan blue (Merck, Milan, Italy) dissolved at 250 μg/mL in ice cold 0.1 M citrate buffer pH 4.0 was added 1:1 in culture wells (microscopy) or to PBS cell suspension (cytofluorimetry) then the samples have been instantly processed one by one for data acquisition [36]. For each experimental point, a measurement was recorded before and after trypan blue addition. An example of flow cytometry carried out in absence or in presence of trypan blue quenching is shown (Figure 1A2 and Figure 5D).

*Fluorescence microscopy:* Images were acquired through a cooled CCD camera (Sensicam; PCO, Kelheim, Germany) and a 40× objective on an Olympus IX81 microscope (Olympus, Tokyo, Japan), equipped with variable light attenuation system, Lumen 200 (Prior Scientific, Rockland, MA, USA) and analyzed using ImageJ software (NIH, Bethesda, MD, USA) [37]. For each experimental point, a total of at least 100 cells were counted, from different fields of the same culture well, if required. In our experimental system >90% phagocytosis events are associated to granular cells (discriminated from other cell types by morphology in phase contrast microscopy), and the remaining <10% are associated to plasmatocytes. Therefore, we evaluate phagocytosis rate as number of fluorescence-labeled cells (granular cells + plasmatocytes)/total cells × 100.

*Flow cytometry:* phagocytosis of FITC-labeled bacteria was evaluated by flow cytometric analysis using a FACSCalibur^®^ flow cytometer (Becton Dickinson, Mountain View, CA, USA) equipped with an air-cooled argon ion laser (15 mW, 488 nm) and data were processed using CellQuestPro software (Becton Dickinson, Mountain View, CA, USA). Fluorescent emission of FITC was collected through a 530 nm band pass filter and quantitated in arbitrary units based on the mean fluorescence intensity (MFI). For each experimental point a total of 10,000 events were acquired.

### 2.6. Densitometric Analysis of Larvae Color Change

Images of whole larval bodies (16-bit grayscale) were acquired with G:BOX Chemi XT4 system (Syngene, Cambridge, UK) and analyzed using ImageJ software (NIH, Bethesda, MD, USA). Larvae have been immobilized by chilling them on ice for 5 min then the pictures have been taken in uniform white led light and constant acquisition parameters.

### 2.7. Nodule Count

Larval bodies have been frozen at −20 °C after the experimental treatments. Frozen animals were longitudinally and dorsoventrally cut with a blade and the images of the two halves where immediately acquired with a Zeiss Stemi SV1 dissection stereomicroscope at 10× (Zeiss, Jena, Germany) [18].

### 2.8. Cell Cultures of the Human Promyelocytic Cell Line THP1 and Phagocytosis Assay

The human promyelocytic cell line THP1 was cultured at 37 °C in 5% CO_2_ in RPMI medium supplemented with 10% FCS, 1% glutamine and 1% antibiotics. In order to increase the phagocytosis rate in THP1, cells were cultured for 72 hours in medium supplemented with phorbol myristate acetate PMA 0.1 nM (Sigma-Aldrich, Milan, Italy). In this condition, THP1, which growths in suspension, adheres and spreads tightly to cell culture plastic and acquires high phagocytosis potential [38]. The phagocytosis experiments were carried out in 24 multi-well plates on PMA-treated THP1 plated at sub confluence 2 × 10^5^ cells/well. The cells were challenged with 5 × 10^6^ cells/well FITC-labeled bacteria at 37 °C, that is a bacterium/hemocytes ratio of 25:1. After 2.5 h at 37 °C in 5% CO_2_, THP1 cell were analyzed, by fluorescence microscopy or, after detachment by treatment with trypsin/EDTA, by flow cytometry. In both cases the evaluation of phagocytosis was carried out after trypan blue quenching. Phagocytosis of FITC-labeled *X. nematophila* by THP1 cells was also analyzed after opsonization of bacteria with rabbit complement (Sigma-Aldrich, Milan, Italy), at 37 °C for 20 minutes, or after treatment with proteinase K or urea.

### 2.9. Data Analysis

Data obtained from phagocytosis assays (fluorescence microscopy and flow cytometry) as fraction of phagocytic cells p = (number of fluorescent cells)/(number of total cells) were transformed by arcsine square root transformation, these results were then analyzed using a two-way analysis of variance (ANOVA) followed by a Tukey honestly significant difference (HSD) test. Pairs of data sets that have been compared and the numerosity of the samples are specified in the figure legends. In all the cases the data shown in the graphs are mean percentage values + standard deviation. Differences were considered significant at least when *p <* 0.05.

## 3. Results

### 3.1. Heat-Killed *X. Nematophila* Escapes In Vitro Uptake by Hemocytes without Directly Inhibiting In Vitro or In Vivo the Cellular Mechanisms of Phagocytosis

We investigated if dead *X. nematophila* bacterial particles escapes internalization by phagocytes. To this end we studied the uptake of heat-killed, FITC-labeled *X. nematophila* by phagocytes of *G. mellonella*. Heat-killed FITC-labeled *E. coli*, is efficiently internalized in in vitro assays (Figure 1A1,B), while *X. nematophila* avoids internalization (Figure 1A2,B). In fact, a high proportion of hemocytes internalize *E. coli*-FITC (Figure 1A1) and several of these cells display a dotted circular pattern of fluorescent bacterial particles accounting for multiple events of phagocytosis. Therefore, *X. nematophila* neither enter insect phagocytes nor appear to interact/adhere with the cell surface of these cells.

To further clarify the nature of the resistance to internalization of heat-inactivated *X. nematophila* particles, the possibility that this bacterium might inhibit the uptake of *E. coli* was investigated. In vitro cultured hemocytes were pre incubated with heat killed *X. nematophila* 3 hours prior to exposure to FITC-labeled *E. coli* and no significant decrease in the internalization of *E. coli* was observed (Figure 1A3,B). Though, the pre-incubation of in vitro cultured hemocytes with alive *X. nematophila* produced a small but statistically significant decrease in the phagocytosis of *E. coli* (Figure 1A4,B). Thereafter, the effect of a challenge with heat killed or alive *X. nematophila* on the phagocytosis of *E. coli* was further investigated in vivo.

**Figure 2:**
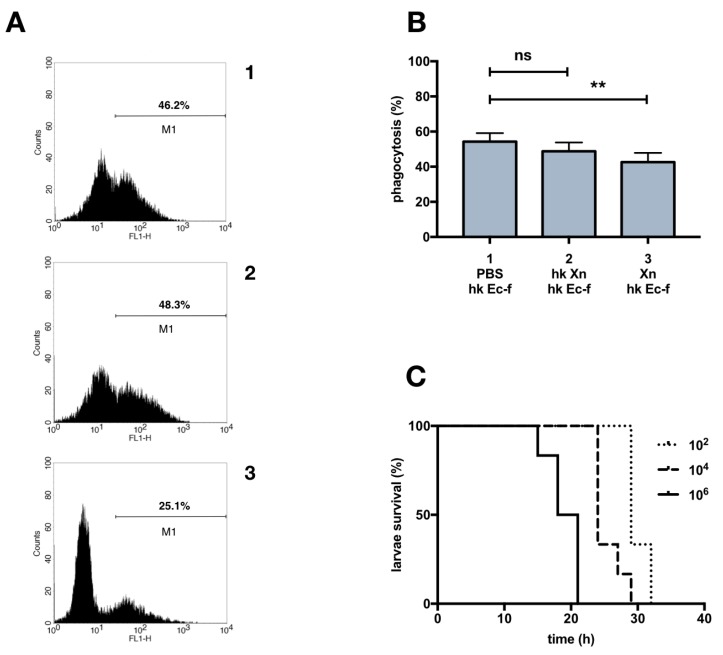
Heat killed *X. nematophila* in vivo does not inhibit *E. coli* phagocytosis. **(A)** double injection experiment, cytofluorimetry of hemocytes extracted from single larva. Caterpillars were injected, with PBS (1), with heat killed *X. nematophila*, 10^7^ particles/larva (hk Xn) (2) or with alive *X. nematophila*, 10^6^ particles/larva (Xn) (3). After 3 hours at 23 °C, larvae were injected with 10^7^ FITC-labeled heat killed *E. coli* (hk Ec-f) and after further 1.5 hours at 23 °C the hemolymph was collected for analysis. M1, percentage of negative cell events; M1, percentage of positive cell events above the auto-fluorescence threshold. **(B)** statistics on cell count microscopy images, same conditions as in A, each experimental point was replicated in five larvae, from three independent experiments (n = 15). **(C)** survival analysis (Kaplan Meier plot) after alive *X. nematophila* injection: 10^2^, 10^4^, 10^6^ bacteria/larva. Statistical analysis was executed on angular transformed data (see material and methods) two-way ANOVA and Tukey HSD test is applied between pairs of data as indicated by horizontal bars (** *p* < 0.01).

Fluorescence microscopy highlights a small but statistically significant decrease in the phagocytosis of FITC-labeled *E coli* when caterpillars were injected with alive, but not with heat killed, *X. nematophila* (Figure 2B), this reduction is supported even by flow cytometry analysis, as shown in a representative experiment (Figure 2A). Moreover, the decrease in *E coli* phagocytosis in the larvae injected with live *X. nematophila* is paralleled by the expected virulent effect as shown in the survival analysis, the Kaplan–Meier plot (Figure 2C). 

### 3.2. Phagocytosis of *X. Nematophila* In Vivo

**Figure 3:**
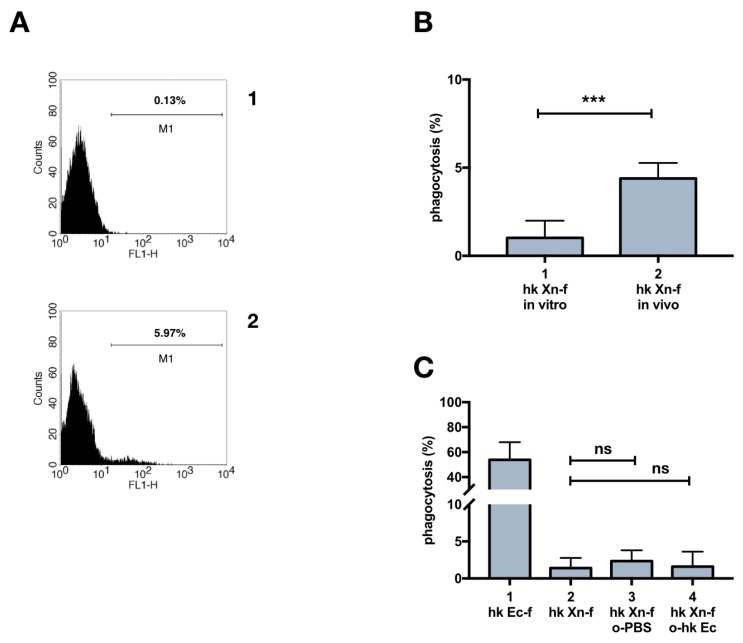
In vivo vs in vitro phagocytosis of heat killed *X. nematophila*, the opsonization of bacteria does not affect its in vitro uptake. **(A)** cytofluorimetry on hemocytes extracted from single larvae: 1, in vitro test, hemocytes cultured, at a calculated bacterium/cell ratio of 25:1, for 2h at 23 °C with heat killed, FITC-labeled *X. nematophila*, (hk Xn-f); 2, in vivo test, hemocytes extracted from single larvae 1.5 h after injection with heat killed, FITC-labeled *X. nematophila*, 10^7^ bacteria/larva (hk Xn-f); M1, percentage of phagocytosis positive cells above the autofluorescence threshold. **(B)** statistics on microscopy images cell counts, each experimental point was replicated in five larvae, it is shown the statistical analysis from three independent tests (n = 15); same experimental conditions as in A. **(C)** in vitro cell counts on fluorescence images: 1, heat killed, FITC-labeled *E. coli* (hk Ec-f); 2, heat killed, FITC-labeled *X. nematophila* (hk Xn-f); 3, heat killed, FITC-labeled *X. nematophila* previously incubated for 30 min at 23 °C in cell-free hemolymph extracted from naïve larvae (hk Xn-f o-PBS); 4, heat killed, FITC-labeled *X. nematophila* previously incubated for 30 min in cell free hemolymph extracted from larvae injected (primed) 24 h in advance with 10^6^ heat killed *E. coli* (hk Xn-f o-hk Ec). Both untreated and opsonized FITC-labeled bacteria were added to wells at a calculated bacteria/cell ratio of 25:1 and after 2.5 h at 23 °C images where acquired. Each experimental point was replicated in five larvae, it is shown the statistical analysis from three independent tests (n = 15). Statistics on B and C are executed on angular transformed data (see material and methods) two-way ANOVA and Tukey HSD test is applied between pairs of data as indicated by horizontal bars (*** *p*<0.001).

We next investigated the phagocytosis activity in vivo after injection of *X. nematophila* in the hemocoel of *G. mellonella* larvae. Surprisingly, under this experimental condition we observed, (Figure 5A1 for a sample image), a small but statistically significant uptake of heat killed bacterial particles (Figure 3B). Nevertheless, even in vivo no uptake of *X. nematophila* was detected in a high proportion of caterpillars (phagocytosis rate in vitro 11/15 larvae 0%, 4/15 larvae ≤2%; phagocytosis rate in vivo 5/15 larvae, 0% 10/15 ≤5%). Similar results were obtained by flow cytometry analysis (Figure 3A), in a representative comparison between in vitro and in vivo experimental settings, a tiny population of phagocytizing hemocytes was detected in vivo (5.9%) but not in vitro (0.1%). 

The in vitro procedure avoids the contact between bacteria and hemolymph borne factors. In order to verify a possible role of insect opsonins in the observed in vivo uptake of *X. nematophila*, fluorescent dead *X. nematophila* particles were challenged with cell-free hemolymph before incubation with hemocytes for the in vitro phagocytosis assay. The results depicted in (Figure 3C) suggest that the contact between entomopathogenic bacteria and host fluids does not rescue in vitro phagocytosis; FITC-labeled *X. nematophila* was pre-treated with pooled hemolymph from naïve or from dead *E. coli* primed caterpillars with similar results. In other words, the absence of bacterial internalization in vitro (shown in Figure 1) could not be simply explained by lack of opsonization. 

### 3.3. Immune System Priming with Heat-Killed *E. Coli* and Effects on *X. Nematophila* Phagocytosis 

We tried to increase the rate of *in vivo* phagocytosis in order to unravel the mechanisms that make the bacterial particle of this entomophatogenic microorganism so difficult to ingest. In principle it is possible to fulfil this task following two different approaches: (a) modifying host physiology, the use of immunomodulatory drugs or other types of treatment, in vivo or in vitro, aimed to implement the phagocytic potential of hemocytes; (b) modifying the bacterium, to reshape the cell surface of *X. nematophila* by biochemical means. As a first attempt to change the host physiology, we investigated if the phagocytosis rate of dead *X. nematophila* may be increased by a previous challenge with dead bacteria, (priming). We used heat killed *E. coli* for the priming, since *X. nematophila*, even dead, is regarded as strongly immunosuppressive in Lepidoptera larvae. Therefore, we challenged *Galleria* larvae with various amounts (tenfold dilutions from 10^6^ to 10^2^ bacteria / larva) of heat-killed *E. coli* 24 h before injection of FITC-labeled *X. nematophila*.

After the priming we did not observe any increase of phagocytosis of FITC-labeled *X. nematophila* above the basal level (Figure S2).

### 3.4. Changes in the Appearance (Darkening) of the Caterpillar Body after *X. Nematophila* Infection, Antagonistic Effect of Immunomodulatory Drugs

**Figure 4:**
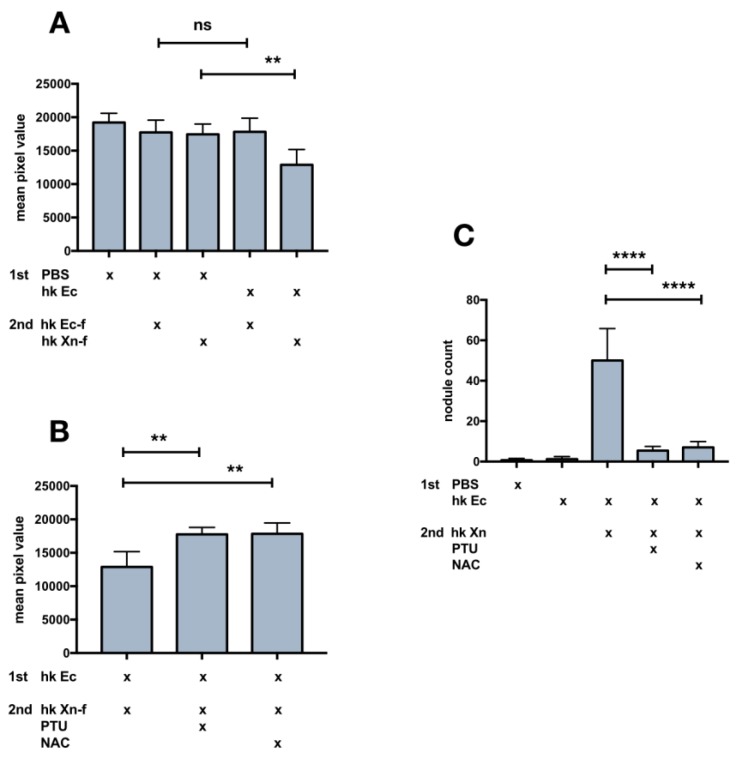
Darkening of larval body and granule count in caterpillars injected with heat killed *X. nematophila*, maintenance of the original color after co-treatment with immunomodulatory drugs. The larvae underwent a cycle of two injections and whole body brightness was measured from images acquired in controlled condition. 1st injection (priming) was carried out 24 hours before the 2nd injection then images are acquired 3 hours after 2nd injection. Five larvae/treatment replicated three times n = 15. (**A**) 1st injection, PBS or 10^6^ heat killed *E. coli* (hk Ec); 2nd injection, 10^7^ heat killed *E. coli* (hk Ec-f) or heat killed *X. nematophila* (hk Xn-f) both FITC-labeled. (**B**) 1st injection, hk *E. coli* (hk Ec); 2nd injection, FITC-labeled heat killed *X. nematophila* (hk Xn-f), FITC-labeled heat killed *X. nematophila* resuspended in PBS containing PTU 5 mM (PTU) or FITC-labeled heat killed *X. nematophila* resuspended in PBS containing NAC 5 μg/μL (NAC). (**C**) nodule count, same experimental condition as A and B, freezed larvae are cut longitudinally, the count is the sum of the dark melanization granules visible in the two halves. Statistics are executed on angular transformed data (see material and methods) two-way ANOVA and Tukey HSD test is applied between pairs of data as indicated by horizontal bars (** *p* < 0.01 ****, *p* < 0.0001).

An interesting finding revealed in the experiments described in the previous result section [3,4] was the darkening of caterpillars few minutes after injection of heat-killed *X. nematophila.* This outcome was observed after the challenge with 10^6^, heat-killed *E. coli* (priming procedure) (Figure 4A), and it is not observed in PBS injected larvae (control). Moreover, the ability of heat-killed *X. nematophila* to produce browning in insect hosts was observed irrespectively from surface bound FITC (Figure S3). We explained the darkening of the caterpillars as the likely outcome of over activation of the pro-PO system. Consequently, we tried to counteract the putative over activation of melanization by co-injecting heat-killed *X. nematophila* with PTU, a well-established competitive inhibitor of the phenoloxidase enzyme. The co-injection prevents the darkening of the larval body (Figure 4B). Caterpillars retained the original light color, amazingly, even after co-injection of dead *X. nematophila* and N-acetyl-cysteine NAC (Figure 4B). Finally, the darkening is paralleled by the formation of melanized nodules in the body cavity of caterpillars and both PTU and NAC counteract this outcome (Figure 4C).

### 3.5. Effects on Host Physiology of Immunomodulatory Drugs 

**Figure 5:**
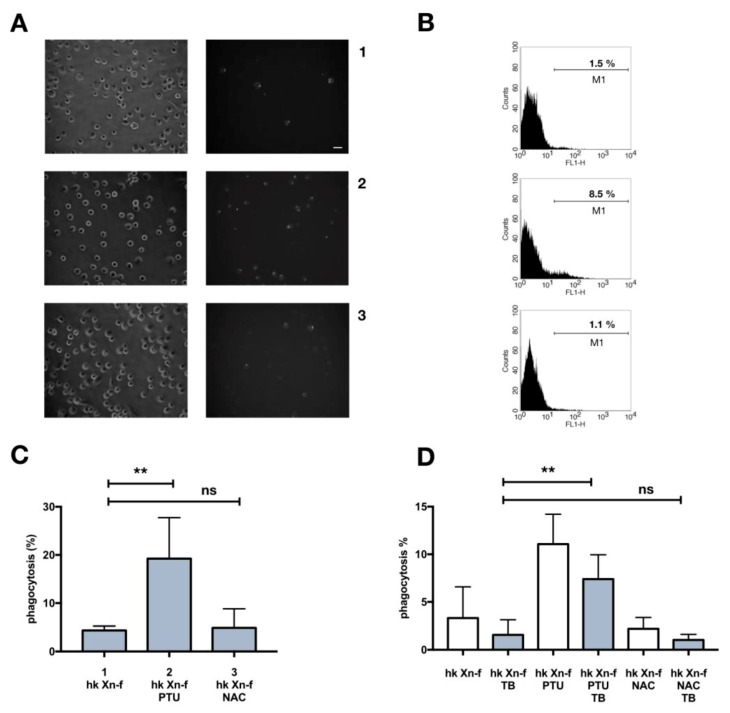
In vivo phagocytosis of *X. nematophila* in larvae co-injected with immunomodulatory drugs. (**A**) bright field (left) and fluorescence (right) images, each pair is from the same field of hemocytes cell cultures. 1, heat killed, FITC-labeled *X. nematophila* (hk Xn-f); 2, heat killed, FITC-labeled *X. nematophila* resuspended in PBS containing PTU 5 mM (hk Xn-f PTU); 3, heat killed, FITC-labeled *X. nematophila* resuspended in PBS containing NAC 5 μg/μL (hk Xn-f NAC). 10^7^ bacteria/larva were injected, bleeding of the caterpillars was carried out after 1.5 h incubation at 23 °C. Fluorescence images are acquired in presence of trypan blue. Calibration bar 20 μm. (**B**) cytofluorimetric analysis on the same cells and conditions as in A. M1, percentage of positive cell events above the autofluorescence threshold. (**C**) cell count statistics from microscopy images, data points obtained from five different larvae for each treatment replicated in three different experiments (n = 15); same conditions as in A. (**D**) statistics on cytofluorimetry, single data point are obtained by different larvae form three different experiments (n = 6); grey bars are duplicate samples, the same shown in empty bars, but with trypan blue (TB) added to the sample right before acquisition. Statistics are executed on angular transformed data (see material and methods) two-way ANOVA and Tukey HSD test is applied between pairs of data as indicated by horizontal bars (** *p* < 0.01).

**Figure 6:**
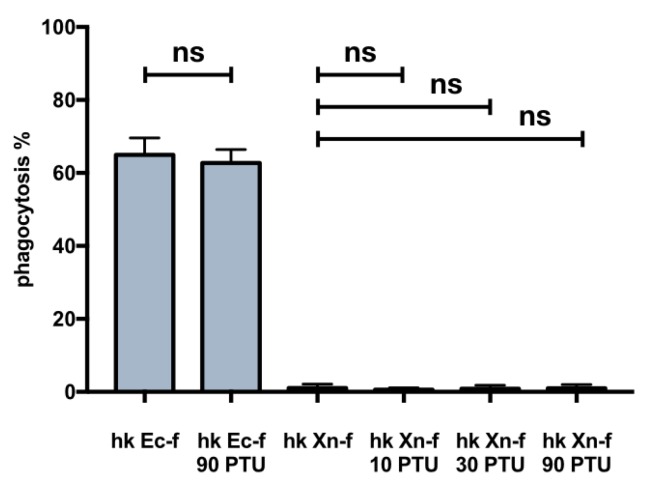
PTU in vitro does not affect phagocytosis of both *E. coli* and *X. nematophila*. Cell count statistics on images acquired from cultured hemocytes, PTU (values represent μM) added to the cell cultures 30 min before incubating with: heat killed, FITC-labeled *E. coli* (hk Ec-f) or heat killed, FITC-labeled *X. nematophila* (hk Xn-f). Hemocytes have been incubated with bacteria for 2.5 h at 23 °C before the observation. Statistics are executed on angular transformed data (see material and methods) two-way ANOVA and Tukey HSD test is applied between pairs of data as indicated by horizontal bars.

Since the priming tests did not increase phagocytosis, and the PTU or NAC “cure” avoided the darkening of the larvae, we assessed if the co-injection of PTU or NAC in naïve larvae could increase the low basal rate of heat killed *X. nematophila* phagocytosis. PTU, but not NAC, induced an increase in phagocytosis, as assessed in a co-injection experiment, by both fluorescence microscopy (Figure 5A,C) and flow cytometry (Figure 5B,D). Moreover, the analysis of flow cytometry conducted before and after trypan blue addition, (Figure 5D) indicates that the increase in the fluorescence signal associated to hemocytes in caterpillars co injected with *X. nematophila* and PTU is mostly due to bona fide phagocytosis. Therefore, we carried out an in vitro assay of phagocytosis on hemocytes freshly separated in Mead buffer and cultured in presence or in the absence of PTU to verify in our experimental model a possible direct effect on phagocytosis of this PO inhibitor. The results indicate neither in vitro increased phagocytosis above the basal level of *X. nematophila* in hemocytes cultured in the presence of PTU nor decreased phagocytosis of *E. coli* in hemocytes cultured in the presence of PTU (Figure 6).

### 3.6. Biochemical Modification of *X. Nematophila* Cell Surface, Effects on In Vitro and In Vivo Phagocytosis

**Figure 7:**
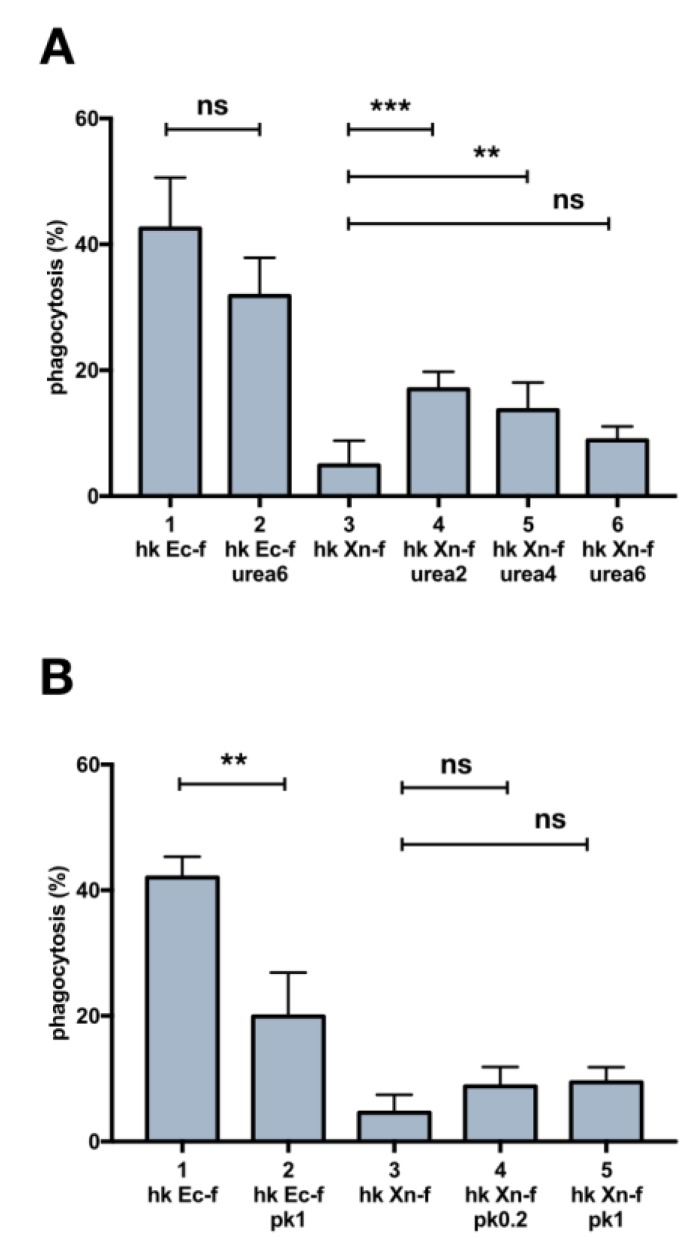
The modification of bacteria cell surface by treatment with urea and proteinase K affects in vivo phagocytosis. (**A**) cell count statistics on images acquired from hemocytes extracted 3h after injection of heat killed, FITC-labeled bacteria treated in different ways: 1, heat killed, FITC-labeled *E. coli*; 2, heat killed, FITC-labeled *E. coli* treated with urea 6 M; 3, heat killed, FITC-labeled *X. nematophila*; 4,5,6 heat killed, FITC-labeled *X. nematophila* treated with different urea concentrations (2 M, 4 M, 6 M) for 10 minutes at 23 °C. **(B)** same protocol as in A but bacteria are exposed to enzymatic treatment: 1, heat killed, FITC-labeled *E. coli*; 2, heat killed, FITC-labeled *E. coli* treated with 1 μg/mL proteinase K; 3, heat killed, FITC-labeled *X. nematophila*; 4,5, heat killed, FITC-labeled *X. nematophila* a treated with two proteinase K concentrations (0.2 μg/mL, 1 μg/mL) for 10 minutes at 23 °C. Statistics are executed on angular transformed data (see material and methods) two-way ANOVA and Tukey HSD test is applied between pairs of data as indicated by horizontal bars (* *p* < 0.1, ** *p* < 0.01).

The resistance to ingestion of *X. nematophila* dead particles is most likely due to the cell surface structural intrinsic features of this microorganism, so we tried to modify the surface of *X. nematophila.* We used two treatments targeting more directly the proteins, urea and proteinase K. We observed a small but significant increase in the phagocytosis rate of FITC-labeled *X. nematophila* pretreated with both urea and proteinase K in vivo (Figure 7A,B) but not in vitro (Figure S4). This increase, at least in bacteria treated with the denaturing agent, was somehow dependent from the concentration being higher after treatment with the lower dose (urea 2M) (Figure 7A,B). Noteworthy both urea and proteinase K treatment, at the higher concentration tested, urea 6M and proteinase K 1μg/mL, significantly decrease the fluorescence signal in *E. coli*.

Most likely this outcome is not due to a real reduction in the phagocytosis of *E coli,* rather it is the result of the peeling of FITC from the cell surface of bacteria as assessed by the presence of FITC molecules in the discarded supernatant of FITC-labeled bacteria after treatment with urea or proteinase K (see material and methods 2.3 for details). Since the brightness of FITC-labeled bacteria results reduced both in *E. coli* and *X. nematophila*, and this experimentally reduces the detection of phagocytosis events, the increased uptake by insect phagocytes of *X. nematophila* after proteinase K or urea treatment is likely underestimated.

### 3.7. Dead *X. Nematophila* Resists Phagocytosis by a Mammalian Macrophage Cell Line

**Figure 8:**
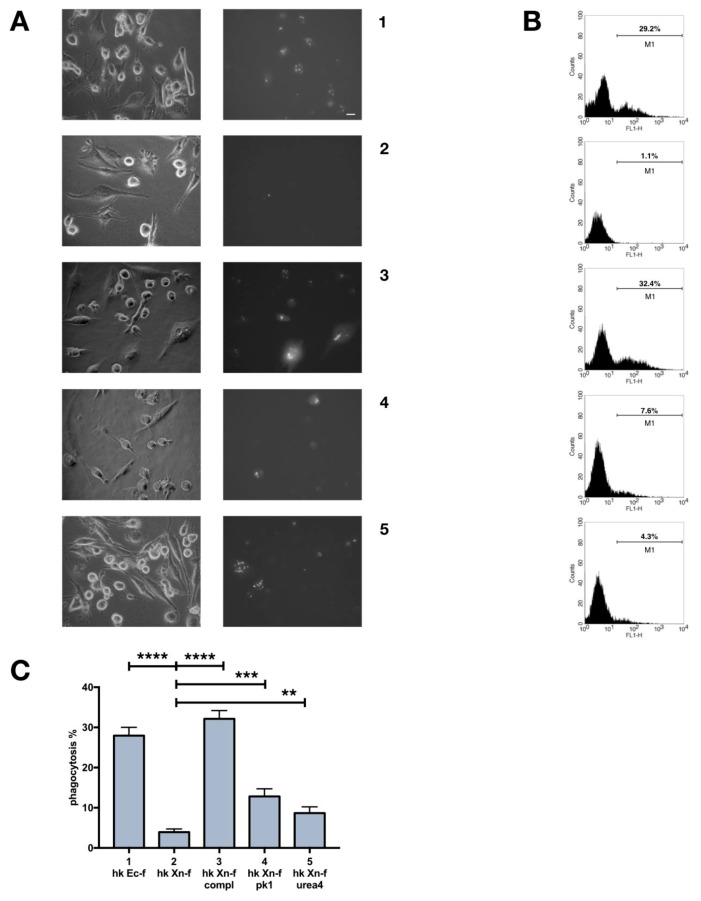
Heat killed *X. nematophila* resists engulfment by human macrophage cell line THP1; recovery of phagocytosis in bacteria opsonized with complement or treated with proteinase K or urea. (**A**) bright field (left) and fluorescence (right) images, each pair is from the same field of THP1 cell cultures. The pro-myelocytic cell line treated with 0.1 nM PMA for 72h prior to phagocytosis test. 1, heat killed, FITC-labeled *E. coli* (hk Ec-f); 2, heat killed, FITC-labeled *X. nematophila* (hk Xn-f); 3, heat killed, FITC-labeled *X. nematophila* treated with rabbit complement 30 min at 37 °C (hk Xn-f compl); 4, heat killed, FITC-labeled *X. nematophila* treated with 1 μg/mL proteinase K for 10 minutes at 23 °C (hk Xn-f pk1); 5, heat killed, FITC-labeled *X. nematophila* treated with urea 4M for 10 min at 25 °C (hk Xn-f urea4). FITC-labeled bacteria were added to cell cultures at a calculated bacterium/cell ratio of 25:1, and 2.5 h later images were acquired. Calibration bar 20 μm. (**B**) cytofluorimetric analysis on the same cells and conditions as in A. M1, percentage of positive cell events above the autofluorescence threshold. (**C**) cell count statistics from images as depicted in A. Each experimental point was replicated in three wells, it is shown the statistical analysis from three independent tests (n = 9). Percentage of phagocytosis is calculated as the fraction of fluorescent cells that display a clear pattern of phagocytosis after trypan blue quenching, multiplied by 100. Statistics is executed on angular transformed data (see material and methods) two-way ANOVA and Tukey HSD test is applied between pairs of data as indicated by horizontal bars (**** *p* < 0.0001; *** *p* < 0.001; ** *p* < 0.01).

Our last concern was to verify the uptake of heat-killed, FITC-labeled *X. nematophila* by phagocytes from an organism very distantly related to insects. As a mammalian phagocyte we used THP1, a human promyelocytic cell line. Interestingly we observed a nearly undetectable internalization of FITC-labeled *X. nematophila* in PMA activated THP1, whereas FITC-labeled *E. coli* was efficiently ingested (Figure 8A1,A2). Then we verified the effect on phagocytosis of FITC-labeled *X. nematophila* after opsonization with rabbit complement. *X. nematophila* was readily engulfed if previously opsonized with complement (Figure 8A3). At last, we studied phagocytosis by THP1 of FITC-labeled *X. nematophila* modified by treatment with urea or proteinase K. The alteration of the cell surface of FITC-labeled *X. nematophila* slightly increase the phagocytosis rate of the bacteria (Figure 8A4,A5). These results were verified both in fluorescence microscopy (Figure 8A,C) and flow cytometry (Figure 8B).

## 4. Discussion

The principal points addressed in this study are briefly summarized below.

*X. nematophila*, even after opsonization, passively resists engulfment by insect hemocytes but it does not actively inhibit the phagocytosis machinery. In comparison to the in vitro experimental setting, in vivo it is observed a small but significant internalization of *X. nematophila*. Co-injection of PTU seemingly enforces *in vivo* phagocytosis of *X. nematophila*, on the other hand *in vitro* tests suggest that PTU does not directly increase phagocytosis of *X. nematophila*. The alteration of *X. nematophila* cell wall by both denaturing and protein degrading agents leads to an increased rate of phagocytosis in vivo. At last, the engulfment of *X. nematophila* in a mammalian professional phagocyte, that is from an organism very distantly related to the insect infection natural host, is nearly undetectable; the opsonization by complement rescues high-rate phagocytosis in this cell line.

*Some pathogenic bacteria in insects and mammals behave akin to X. nematophila.* In our study *X. nematophila* seems to be resistant to opsonin independent internalization by phagocytes without damaging the cellular machinery of phagocytosis. As suggested for *Photorhabdus luminescens* [39], *X. nematophila* does not inhibit *E. coli* phagocytosis; so, its presence in the host hemolymph does not impair the phagocytosis machinery [27]. Instead, in mammals it has been reported an active inhibition of opsonin independent phagocytosis [14,15,40]. Moreover, in order to disrupt the signal transduction pathways of phagocytosis, pathogenic bacteria or fungi of mammals often adhere tightly to the cell surface of phagocytes [14,41,42]; this does not seem to be the case with *X. nematophila*, in fact we rarely observe association of this bacteria to the cell surface of *G. mellonella* hemocytes. Therefore, our results suggest a lack of physical interaction between the *X. nematophila* cell wall and the cell membrane of phagocytes, in other words bacteria somehow remain unrecognized. Although not common, in mammals some pathogens, for example some fungi, passively escape phagocytosis due to an overall ineffective contact to phagocytes. The resistance to internalization in these cases is accomplished because these pathogens are enveloped by a cell coat hiding the PAMPs ligands of phagocytic receptors [43,44] or because these pathogens feature shape/dimensions not well suited for engulfment [14,45]. A similar situation has been reported also in insects; fungal parasites such as *Metarhizium anisopliae* develop during a host’s infection so-called hyphal bodies lacking a thick cell wall and hence PAMPs required for recognition by *G. mellonella* hemocytes [46]. In this regard, our preliminary results with agents like urea or proteinase K that scratch the cell wall of *X. nematophila* suggest that this bacterium results in being unrecognized by phagocytes because the ligand of phagocytic receptors are buried, hidden or disguised in the cell wall. In most cases mammalian pathogens escape or inhibit phagocytosis because they are covered by a capsule [14,15,40] but some exceptions have been studied as well [47]. Although bacteria of the genus *Xenorhabdus* are not covered by a capsule [31] they may present a pronounced hairy surface due to the presence of dense pili/fimbriae structures [30,31] that may account for both disguise of the ligand of phagocytic receptors and increased effective volume hampering internalization by phagocytes. Experiments are underway in order to further explore these issues.

*Disparity observed comparing in vitro versus in vivo phagocytosis.* As a matter of fact, the difference between in vitro and in vivo *X. nematophila* phagocytosis is slight small, although statistically significant, and moreover, it increases after co-injection with PTU. Instead, our in vitro experiments suggest that PTU does not make *X. nematophila* phagocytosis more efficient so, PTU does not produce a direct effect on the phagocytosis mechanism of *X. nematophila*. Seemingly, in conflict with our results, some evidence supports the notion that in insects the melanization cascade aids the phagocytosis process [4], in this frame PTU co-treatment is expected to decrease rather than increase phagocytosis. Indeed, this was the cases as observed in vitro [48,49] but with some caveats [48]; moreover, to the best of our knowledge, the effect of PTU on in vivo phagocytosis has not been investigated so far. Moreover, in insects the connection between phagocytosis and melanization cascade is controversial at the present, since species even from closely related taxa, display different functional outcomes [50]; in fact, generalizations about insect immunity are sometimes problematic due to the great diversity of these organisms.

In conclusion, our results indicate that PTU co-treatment does not increase phagocytosis efficiency of *X. nematophila* but somehow makes more efficient the sampling of phagocytosis events in the hemolymph after the bleeding of caterpillars. A possible explanation is that PTU restrains the over activation of melanization cascade that entrap phagocytosing hemocytes within caterpillars. Our results suggest that, for reasons that have to be determined, heat-inactivated *X. nematophila* may be internalized by phagocytes exerting their function in vivo but not by a host’s phagocytes placed in an in vitro experimental setting. Another issue worth of further study is the apparent over-activation of the pro-PO system in caterpillars primed with heat killed *E. coli* and challenged with dead *X. nematophila.* This finding may be explained because, besides constitutive expression, proPO system is also quantitatively inducible. In fact, somewhat consistent with our result, it has been reported that the activity of phenoloxidase is higher in *G. mellonella* larvae primed with bacteria [51]. At last, both PTU and NAC co-treatments maintain the original body color in caterpillars primed with dead *E. coli* and challenged with dead *X. nematophila*; whereas, only PTU produces an in vivo increased phagocytosis of *X. nematophila.* In this regard, PTU is an inhibitor of phenoloxidase enzyme, whereas NAC is a well-known scavenger of ROS [52] but also a putative melanization inhibitor along with glutathione and other reducing agents [53,54]. Experiments are underway to address these topics in more detail. 

*Xenorhabdus nematophila resists phagocytosis by mammalian professional phagocytes.* The last point is the finding that the engulfment of *X. nematophila* by a phagocyte from the human species is nearly undetectable; moreover, the opsonization of *X. nematophila* by complement rescues high-rate phagocytosis. This evidence suggests that the cell surface of *X. nematophila* is somehow masked or disguised from immune recognition by mammalian phagocyte receptors in a way that is evolutionarily conserved over eons of divergent evolution between Arthropoda and Vertebrates. Moreover, the cell wall binding of *X. nematophila* with activated complement fragments, most likely covalently bound C3b, results in being “dominant” over the lack, or rather the disguise, of suitable ligands for immune recognition on cell surface. Furthermore, and probably more interesting, the study of *X. nematophila* on mammalian phagocytes may be experimentally useful since many more tools and reagents are available in mammalian model species than in insects. This fact would permit the establishment of *X. nematophila* as a model pathogenic bacterium for the study of escape from phagocytosis even in mammalian immunology.

## 5. Conclusions

The principal element of novelty of our study is the finding that dead *X. nematophila* escapes engulfment by insect hemocytes in vitro, but not in vivo. 

Moreover, the difference between in vitro and in vivo experimental conditions further increases after co-injection of bacteria with PTU; as a matter of fact, our data indicate that the inhibitor of the pro-PO system does not exert a direct effect on phagocytosis. Finally, we have demonstrated that the difference between in vitro and in vivo was not due to the absence of opsonins in the in vitro experimental setting. To the best of our knowledge a significant in vivo phagocytosis of an established bacterial pathogen known to efficiently escape phagocytosis in vitro tests has not been reported previously.

## Supplementary Materials

The following are available online at https://www.mdpi.com/2075-4450/10/9/263/s1: Figure S1: fluorescence measurement in the supernatant of FITC-labeled bacteria after treatment with proteinase K or urea, Figure S2: In vivo *X. nematophila* phagocytosis after priming with different amounts of heat killed *E coli*, Figure S3: Darkening of larval body: effect of FITC-labeled versus non labeled *X. nematophila*, Figure S4: In vitro phagocytosis of FITC-labeled *X. nematophila* is not influenced by treatment of bacteria with proteinase K or urea.
