# Supplementary material for: In Vivo Effects of A Pro-PO System Inhibitor on the Phagocytosis of Xenorhabdus Nematophila in Galleria Mellonella Larvae
Source: Insects. 2019 Aug 22;10(9):263. doi: 10.3390/insects10090263 (PMC6780223; doi:10.3390/insects10090263)
Supplement: Supplementary file 1 [file insects-10-00263-s001.zip › suppl fig captions.docx]

**Supplementary Figure 1 fluorescence measurement in the supernatant of FITC-labeled bacteria after treatment with proteinase K or urea.**

Heat killed and FITC-labeled bacteria *E. coli* (**A**) *X. nematophila* (**B**) were treated with the indicated amounts of proteinase K (μg/mL), urea (M) or left untreated (contr). After centrifugation, properly diluted supernatants were analyzed for FITC content with a spectrofluorometer. The results are expressed as the ratio between the fluorescence intensity at each experimental point and the maximal value corresponding to the pk 1 μg/mL treatment.

**Supplementary Figure 2 in vivo *X. nematophila* phagocitosys after priming with different amounts of heat killed *E coli*.**

Cell count statistics from microscopy images, data points obtained from five different larvae for each treatment (n=5). 1st injection, PBS (0) or increasing amounts from 10^2^ to 10^6^ of heat killed *E. coli* (hk Ec); 2nd injection, heat killed FITC-labeled *X. nematophila*, 10^7^ cells/larvae. 1st injection (priming) was carried out 24 hours before the 2nd injection, then after 1.5 hours hemocytes were extracted and images were acquired.

**Supplementary Figure 3 darkening of larval body: effect of FITC-labeled vs non labeled *X. nematophila.***

The larvae underwent a cycle of two injections and whole body brightness was measured from images acquired in controlled conditions. 1st injection (priming) was carried out 24 hours before the 2nd injection then images were acquired 3 hours after 2nd injection.

1st injection, 10^6^ heat killed *E. coli* (hk Ec); 2nd injection, 10^7^ heat killed *X. nematophila* (hk Xn) or 10^7^ heat killed, FITC-labeled *X. nematophila* (hk Xn-f). Five larvae/treatment (n=5).

**Supplementary Figure 4 in vitro phagocitosys of FITC-labeled *X. nematophil*a is not influenced by treatment of bacteria with proteinase K or urea.**

Cell count statistics on images acquired from cultured hemocytes: heat killed, FITC-labeled *X. nematophila* (hk Xn-f) have been treated with the modification protocols as explained in Figure 7; proteinase K expressed in μg/mL, urea in M. Hemocytes have been incubated with bacteria for 2.5 h at 23 °C before the observation, n=5.
