# Supplementary material for: In Vivo Effects of A Pro-PO System Inhibitor on the Phagocytosis of Xenorhabdus Nematophila in Galleria Mellonella Larvae
Source: Insects. 2019 Aug 22;10(9):263. doi: 10.3390/insects10090263 (PMC6780223; doi:10.3390/insects10090263)
Supplement: Supplementary file 1 [file insects-10-00263-s001.zip › suppl fig1.pdf]

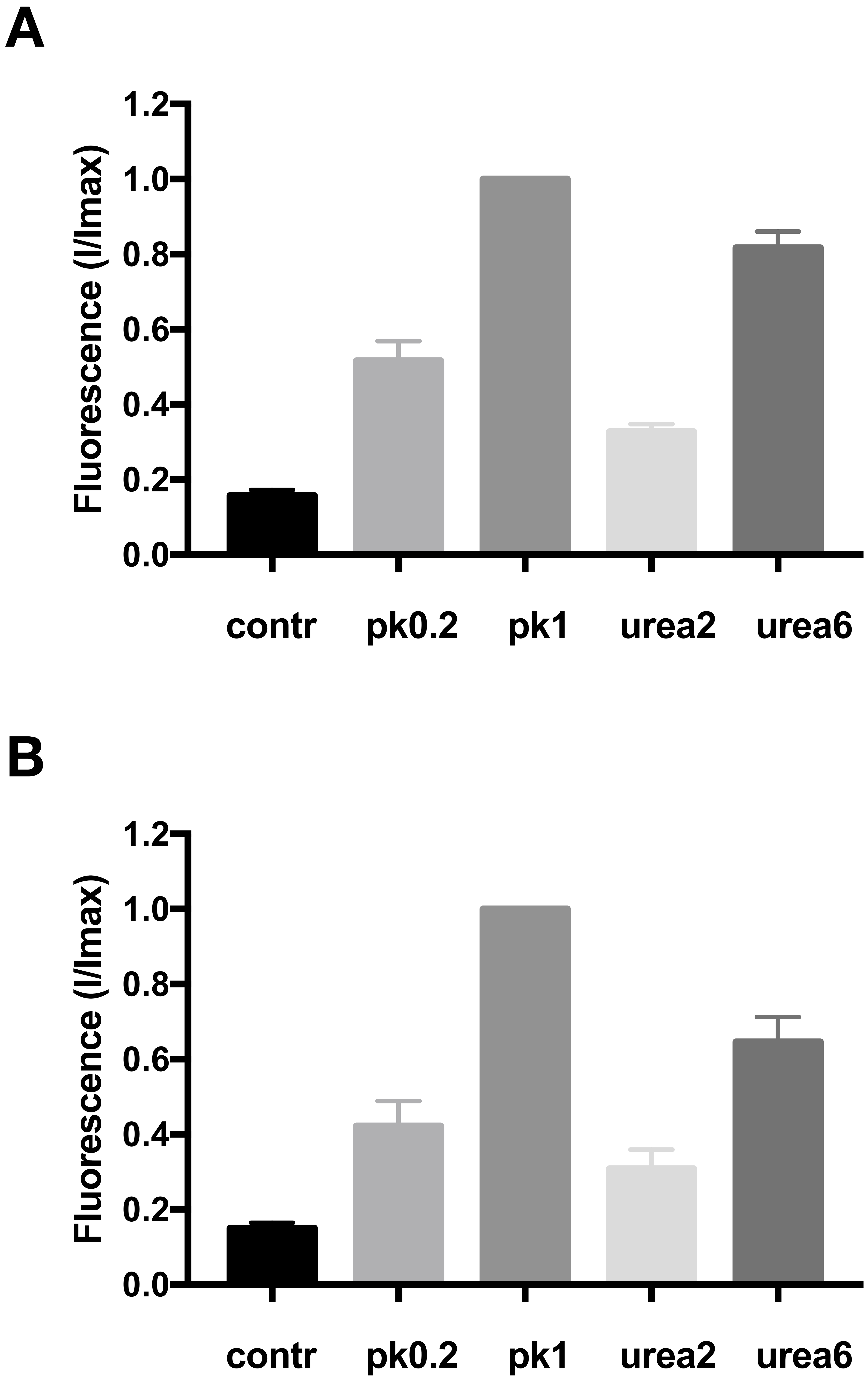

Supplementary Figure 1 fluorescence measurement in the supernatant of FITC-labeled bacteria after treatment with proteinase K or urea.

Heat killed and FITC-labeled bacteria *E. coli* (**A**) *X. nematophila* (**B**) were treated with the indicated amounts of proteinase K (µg/mL), urea (M) or left untreated (contr). After centrifugation, properly diluted supernatants were analyzed for FITC content with a spectrofluorometer. The results are expressed as the ratio between the fluorescence intensity at each experimental point and the maximal value corresponding to the pk 1 µg/mL treatment.
