# Supplementary material for: In Vivo Effects of A Pro-PO System Inhibitor on the Phagocytosis of Xenorhabdus Nematophila in Galleria Mellonella Larvae
Source: Insects. 2019 Aug 22;10(9):263. doi: 10.3390/insects10090263 (PMC6780223; doi:10.3390/insects10090263)
Supplement: Supplementary file 1 [file insects-10-00263-s001.zip › suppl fig3.pdf]

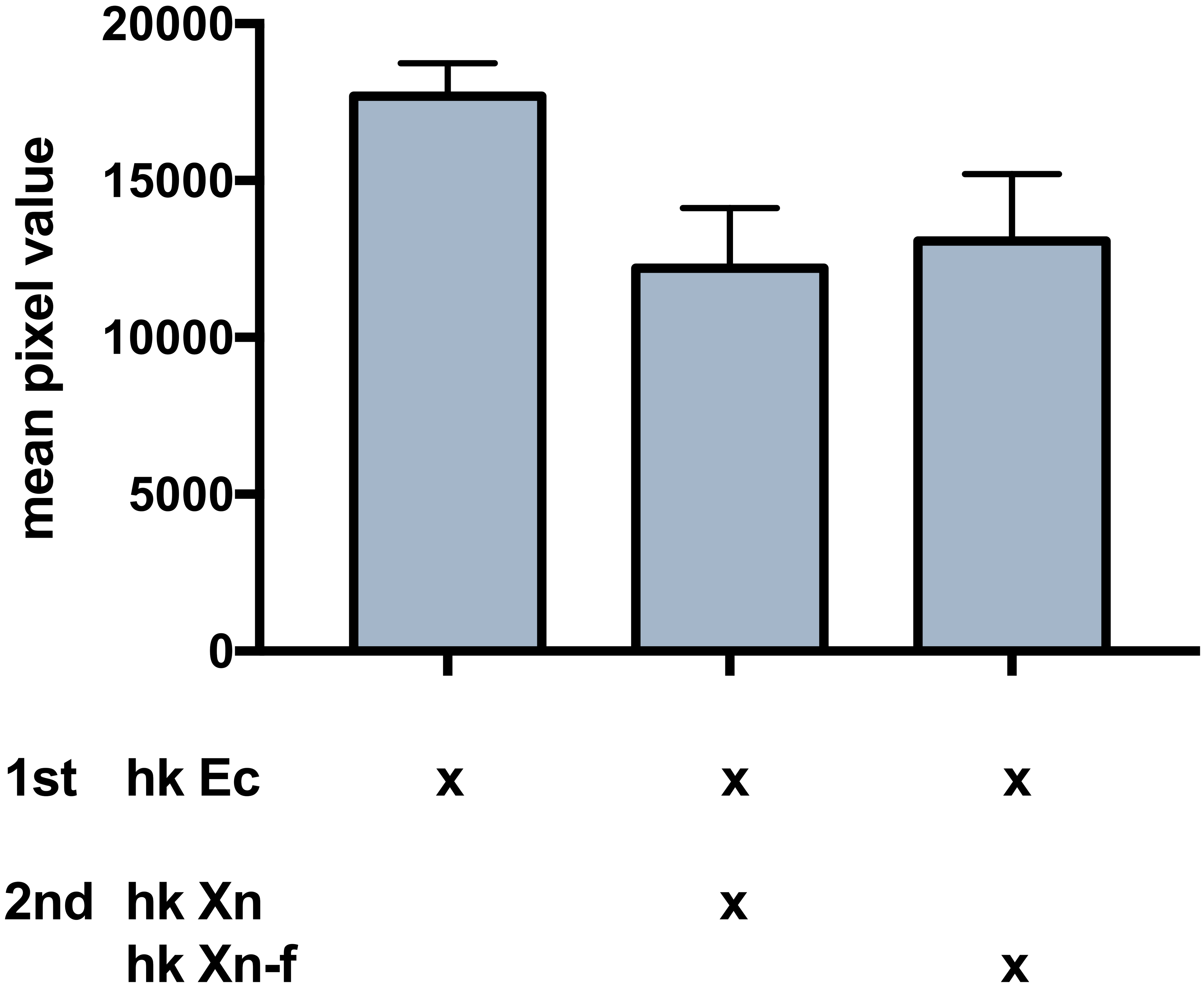

Supplementary Figure 3 darkening of larval body: effect of FITC-labeled vs non labeled *X. nematophila.*

The larvae underwent a cycle of two injections and whole body brightness was measured from images acquired in controlled conditions. 1st injection (priming) was carried out 24 hours before the 2nd injection then images were acquired 3 hours after 2nd injection.
1st injection, 10^6 heat killed *E. coli* (hk Ec); 2nd injection, 10^7 heat killed *X. nematophila* (hk Xn) or 10^7 heat killed, FITC-labeled *X. nematophila* (hk Xn-f). Five larvae/treatment (n=5).
