# Supplementary material for: In Vivo Effects of A Pro-PO System Inhibitor on the Phagocytosis of Xenorhabdus Nematophila in Galleria Mellonella Larvae
Source: Insects. 2019 Aug 22;10(9):263. doi: 10.3390/insects10090263 (PMC6780223; doi:10.3390/insects10090263)
Supplement: Supplementary file 1 [file insects-10-00263-s001.zip › suppl fig2.pdf]

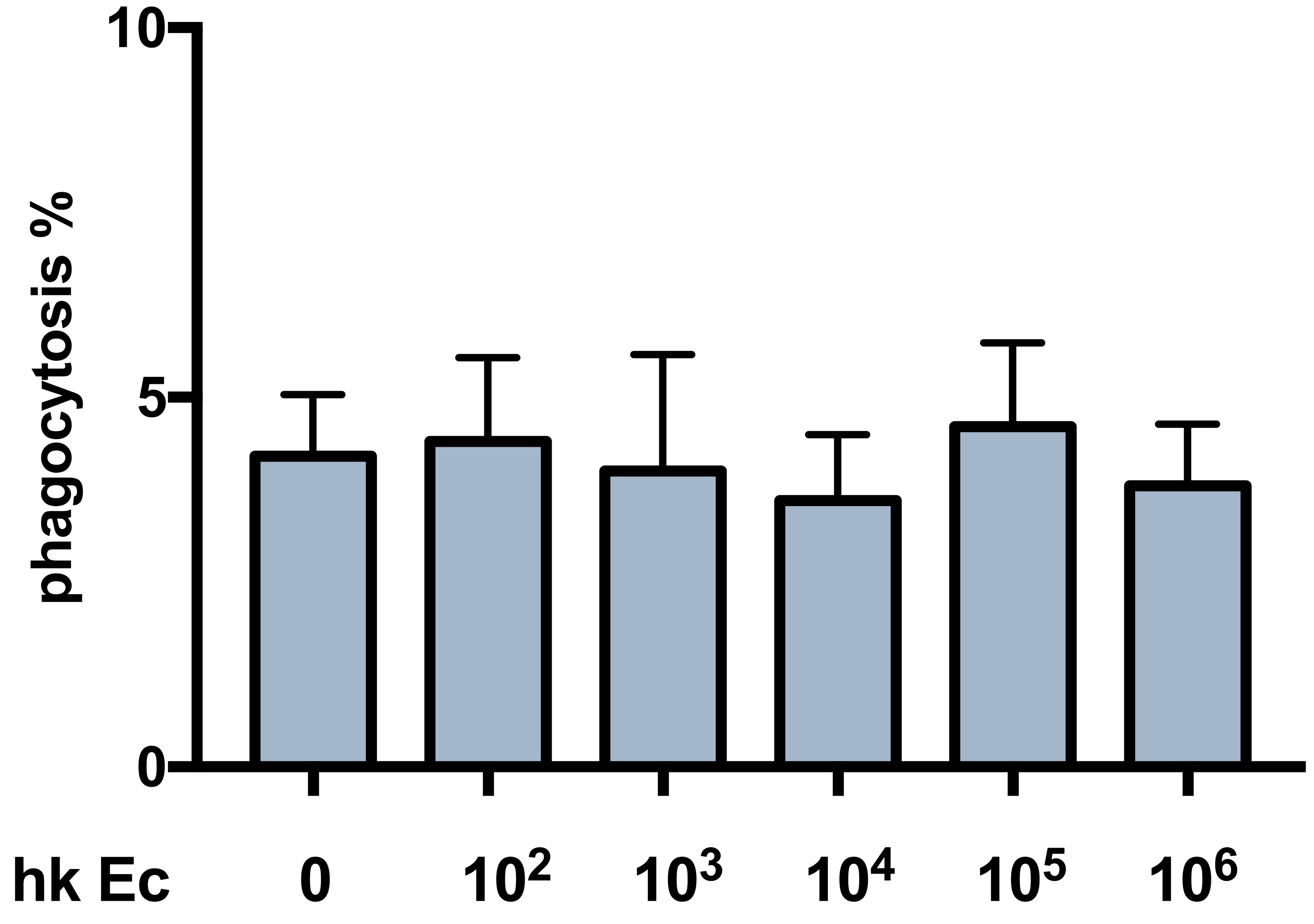

Supplementary Figure 2 in vivo *X. nematophila* phagocitosys after priming with different amounts of heat killed *E coli*.

Cell count statistics from microscopy images, data points obtained from five different larvae for each treatment (n=5). 1st injection, PBS (0) or increasing amounts from 10^2 to 10^6 of heat killed *E. coli* (hk Ec); 2nd injection, heat killed FITC-labeled *X. nematophila*, 10^7 cells/larvae. 1st injection (priming) was carried out 24 hours before the 2nd injection, then after 1.5 hours hemocytes were extracted and images were acquired.
