# Supplementary material for: In Vivo Effects of A Pro-PO System Inhibitor on the Phagocytosis of Xenorhabdus Nematophila in Galleria Mellonella Larvae
Source: Insects. 2019 Aug 22;10(9):263. doi: 10.3390/insects10090263 (PMC6780223; doi:10.3390/insects10090263)
Supplement: Supplementary file 1 [file insects-10-00263-s001.zip › suppl fig4.pdf]

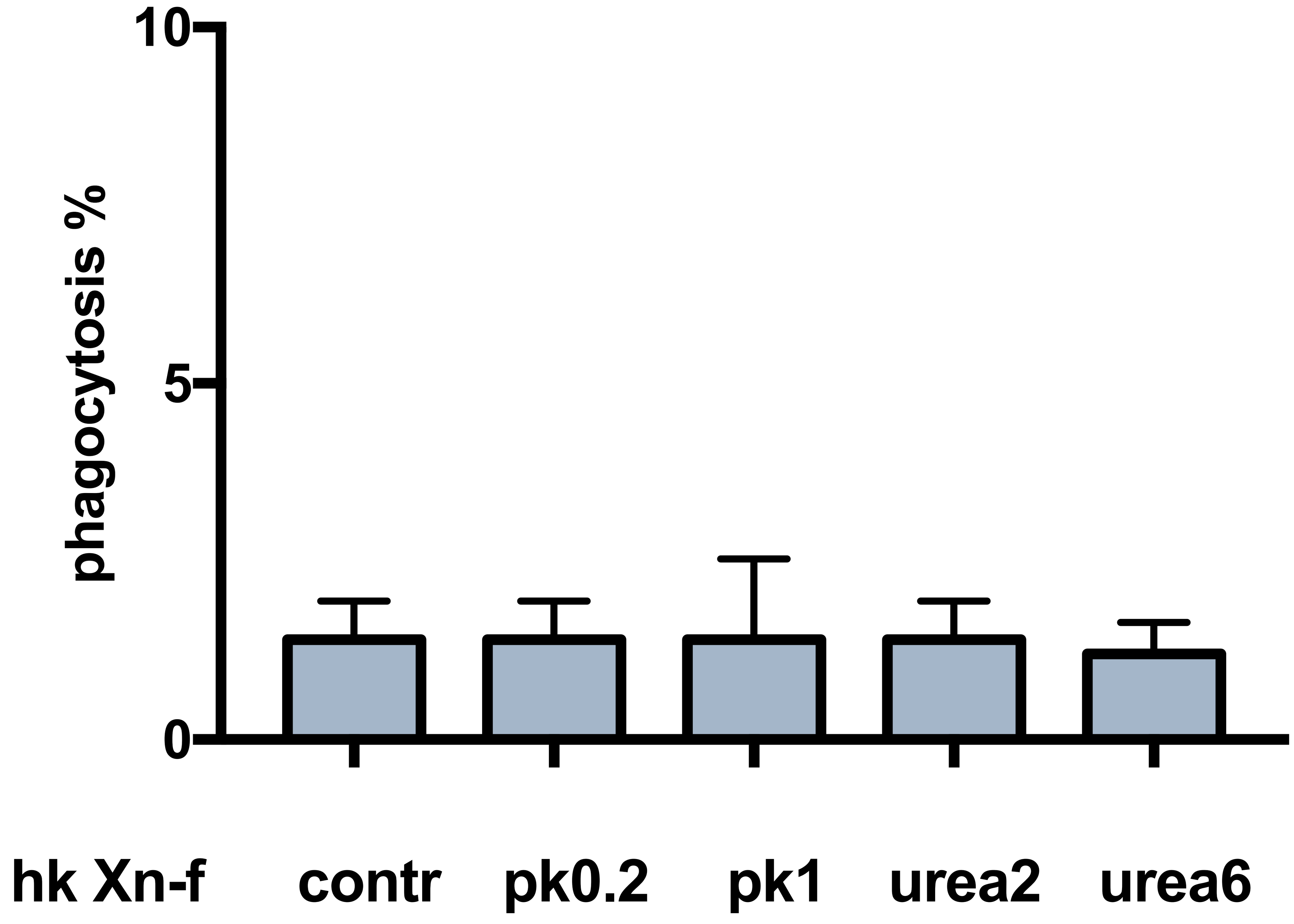

Supplementary Figure 4 in vitro phagocitosys of FITC-labeled *X. nematophil*a is not influenced by treatment of bacteria with proteinase K or urea.

Cell count statistics on images acquired from cultured hemocytes: heat killed, FITC-labeled *X. nematophila* (hk Xn-f) have been treated with the modification protocols as explained in Figure 7; proteinase K expressed in µg/mL, urea in M. Hemocytes have been incubated with bacteria for 2.5 h at 23 °C before the observation. n=5
