# Supplementary figures and images for: In Vivo Effects of A Pro-PO System Inhibitor on the Phagocytosis of Xenorhabdus Nematophila in Galleria Mellonella Larvae
Source: Insects. 2019 Aug 22;10(9):263. doi: 10.3390/insects10090263 (PMC6780223; doi:10.3390/insects10090263)

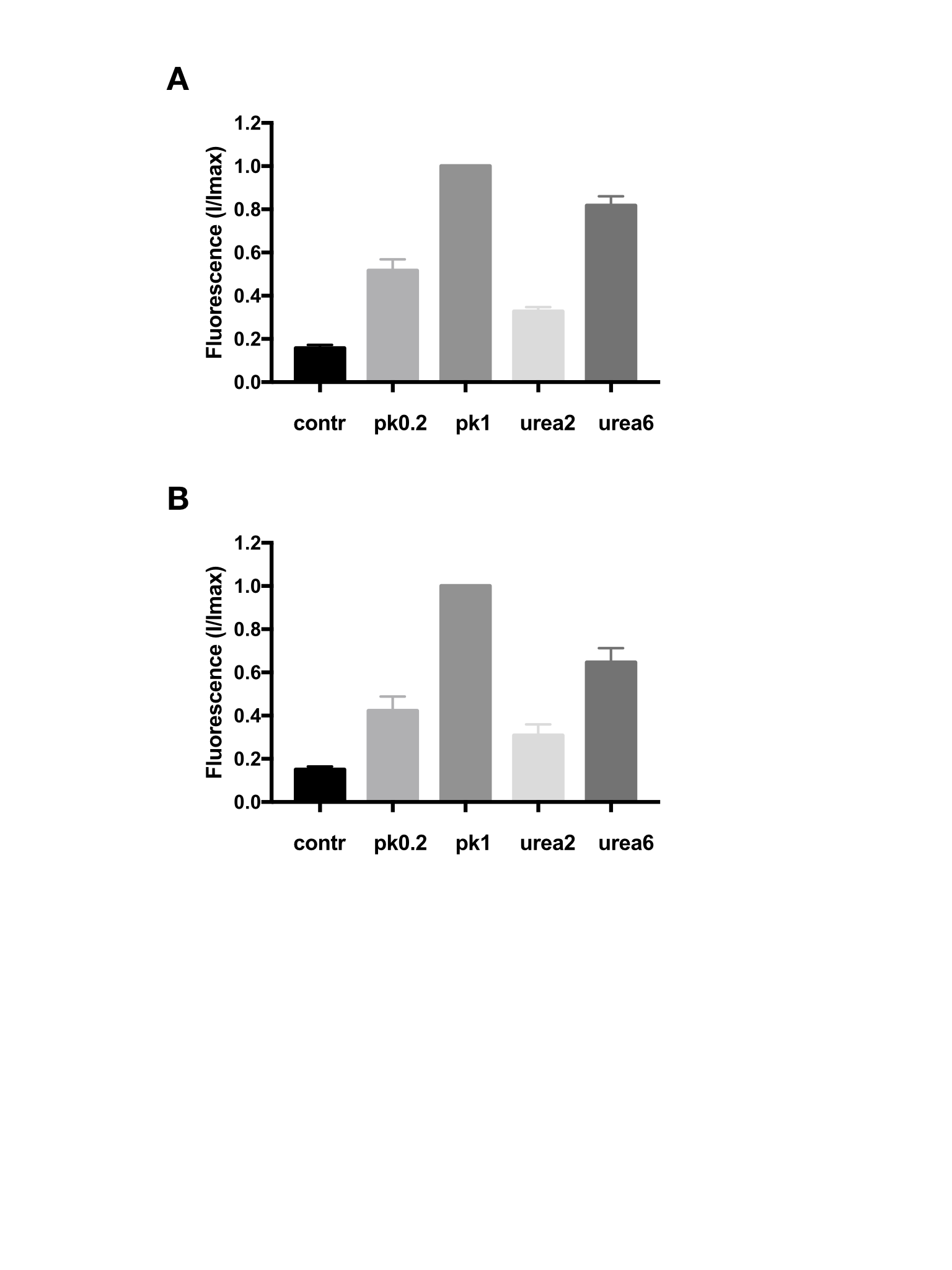

Supplement: Supplementary file 1 [file insects-10-00263-s001.zip › suppl fig1.tiff]

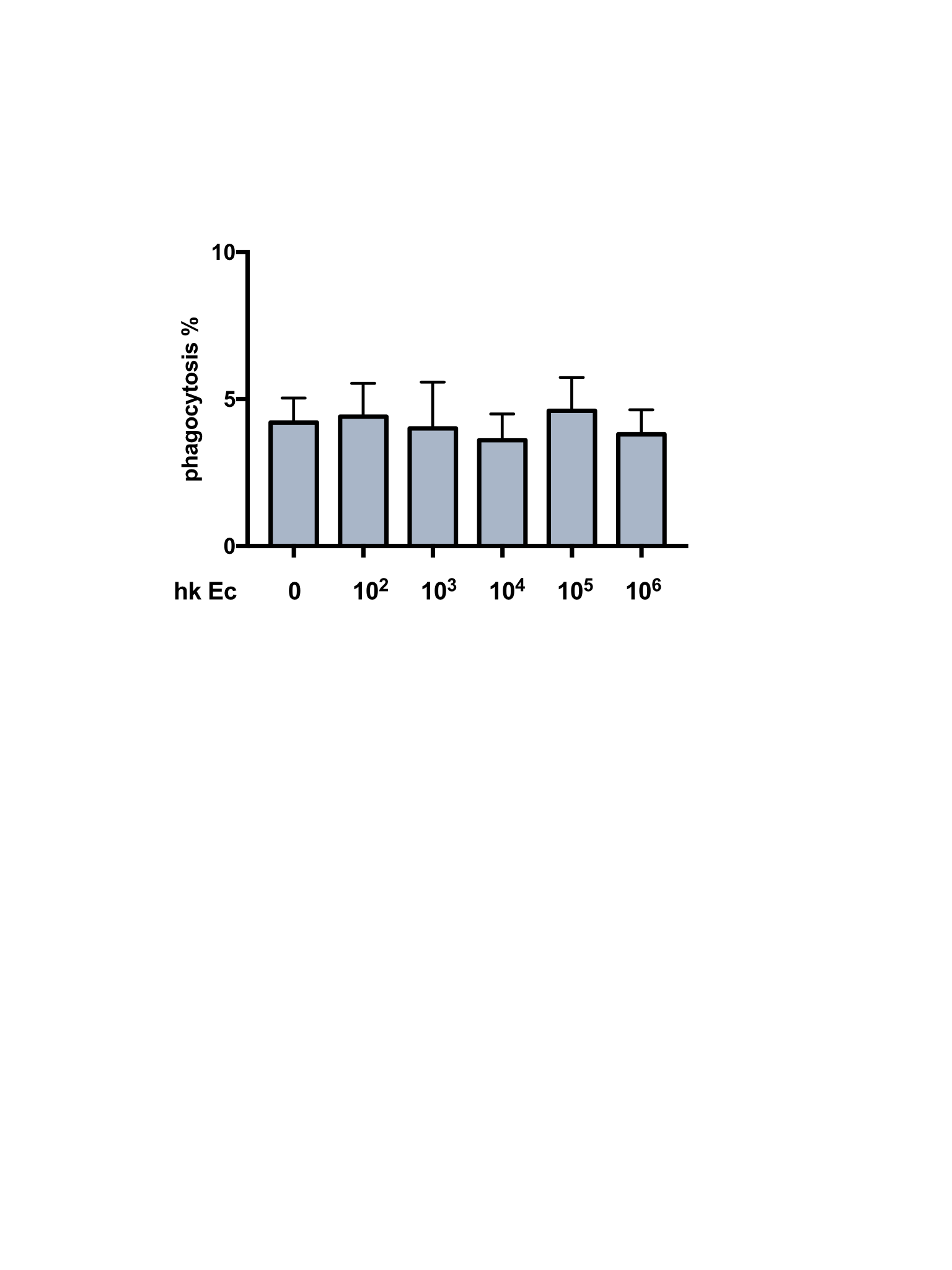

Supplement: Supplementary file 1 [file insects-10-00263-s001.zip › suppl fig2.tiff]

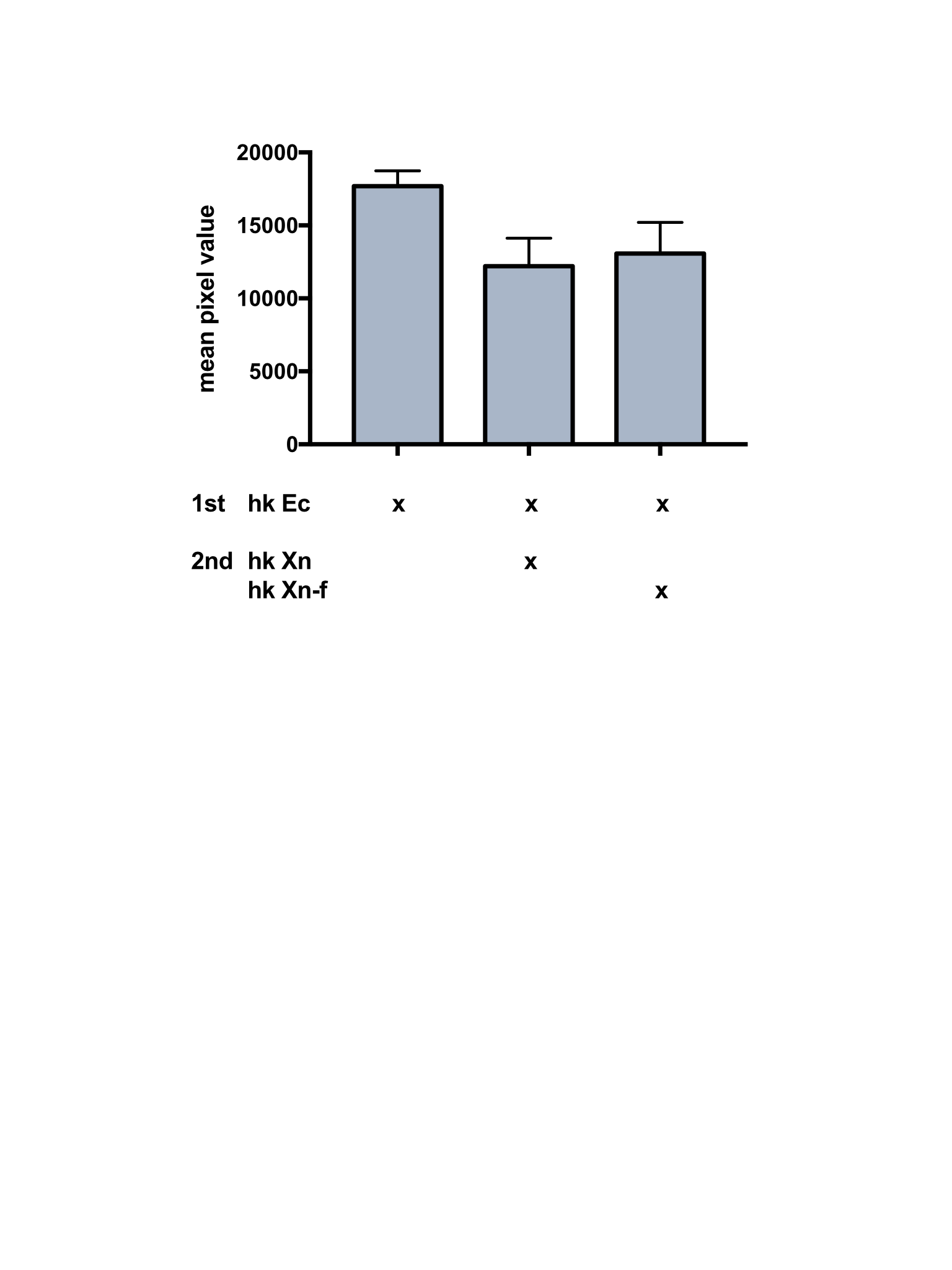

Supplement: Supplementary file 1 [file insects-10-00263-s001.zip › suppl fig3.tiff]

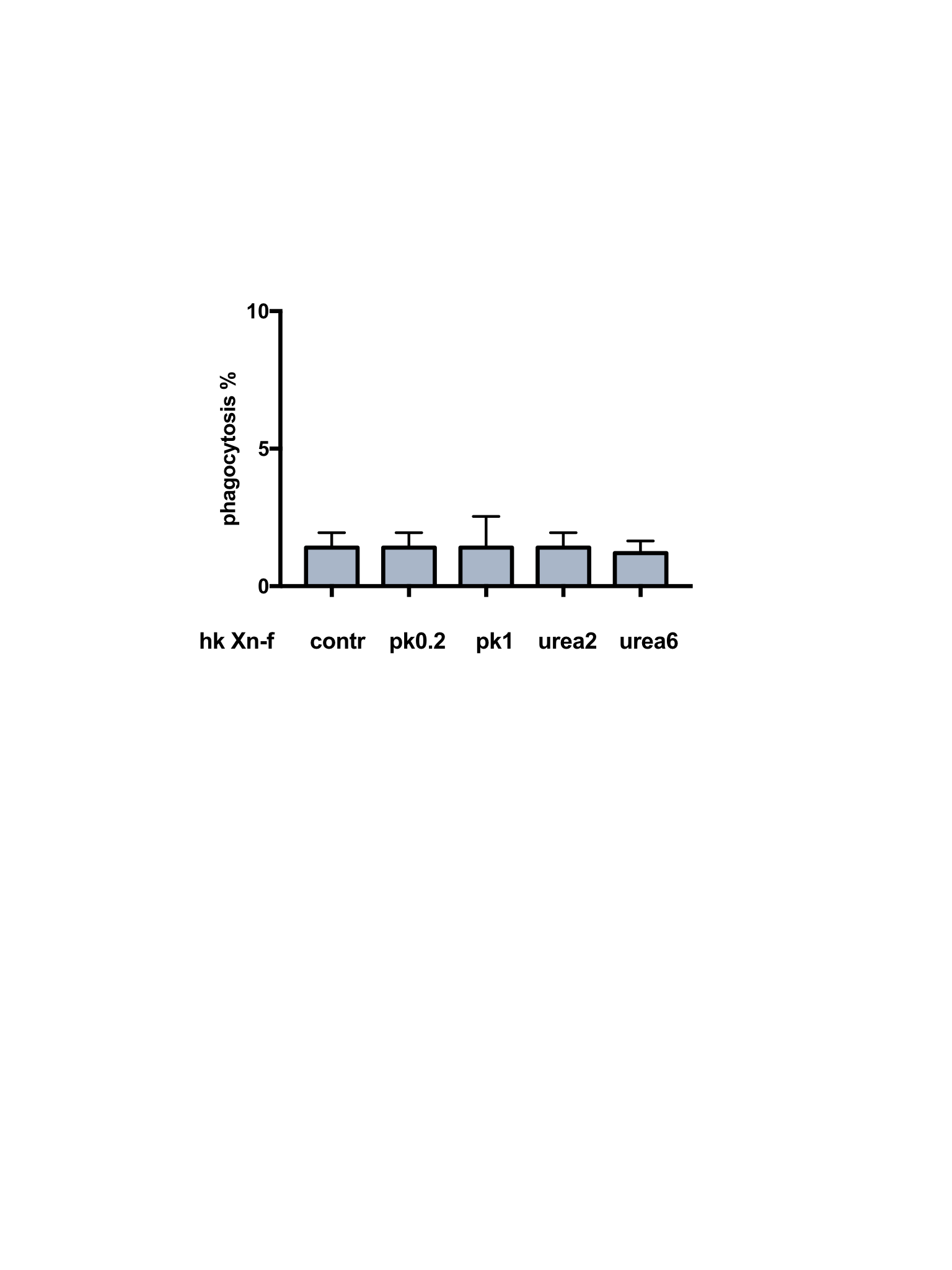

Supplement: Supplementary file 1 [file insects-10-00263-s001.zip › suppl fig4.tiff]
